# Microbially produced flavors and fragrances: An annotated selection of World Wide Web sites relevant to the topics in microbial biotechnology

**DOI:** 10.1111/1751-7915.13961

**Published:** 2021-11-18

**Authors:** Lawrence P. Wackett

**Affiliations:** ^1^ Department of Biochemistry Molecular Biology & Biophysics BioTechnology Institute University of Minnesota St Paul MN 55108 USA

## Microbial biotechnology to produce perfume ingredients

### http://medcraveonline.com/JMEN/JMEN‐02‐00034.pdf

This review article presents a good overview of the molecules and how they are produced microbially.

## Microbial production of scents and flavors

### https://www.sciencedirect.com/science/article/abs/pii/S0958166915001251

This review article focuses on the metabolic pathways involved in the microbial production of aroma and flavor molecules.

## Sweet smell of microbes

### https://cen.acs.org/articles/90/i29/Sweet‐Smell‐Microbes.html

This article in the *Chemical and Engineering News* highlights the emerging transition of the industry from conventional chemical synthesis to microbial fermentation.

## Flavors and fragrances

### https://core.ac.uk/download/pdf/161657099.pdf

This review article focuses on chemical classes of molecules in flavors and fragrances. It discusses why they are important and how they are produced microbially.

## Microbial cell factories for terpenes

### https://www.unboundmedicine.com/medline/citation/28418659/Microbial_Cell_Factories_for_the_Production_of_Terpenoid_Flavor_and_Fragrance_Compounds_

This summary links to an article on microbial production of terpenes. Historically, terpenes are among the most important of the flavor and fragrance molecules. Recent bioengineering of the core terpene biosynthetic pathways has opened up new routes to many important molecules.

## Microbial production of butyl butyrate

### https://koasas.kaist.ac.kr/handle/10203/253991

This research study describes the enzymatic synthesis of butyl butyrate.

## Pyrazines: Flavors and fragrances

### https://onlinelibrary.wiley.com/doi/10.1002/biot.202000064

Traditionally, commercial pyrazines for flavor and fragrance applications have been extracted from plant material. Recent advances make it possible to derive these compounds more inexpensively via microbial fermentations.

## Yeast perfume

### https://www.newscientist.com/article/mg22530113‐600‐would‐you‐wear‐yeast‐perfume‐microbes‐used‐to‐brew‐scent/

In this popular science article, scientists and business people in the flavor and fragrance are interviewed. They paint a picture that the industry is moving increasingly to microbial production of desired aroma and flavor molecules.

## Microbes influencing flavor

### https://microbiologysociety.org/publication/past‐issues/microbes‐and‐food/article/a‐matter‐of‐taste‐using‐microbes‐to‐influence‐flavour‐production.html

In addition to being used to extract pure compounds, microbes have often been added to foods to enhance flavor. This article discusses that point using examples like yogurt and cheese.

## Microbial methyl anthranilate

### https://www.pnas.org/content/116/22/10749

Methyl anthranilate is not a household name, but it is a popular choice in industry to use for providing grape flavoring and for scent in perfumes. This study describes its production in *Escherichia coli* and *Corynebacterium glutamicum*.

## International Flavors and Fragrances

### https://www.encyclopedia.com/economics/economics‐magazines/international‐flavors‐and‐fragrances‐inc

It is worth wading through the advertisements on this site if one is interested in the history and business of a major player in the flavor and fragrance industry.

## Givaudan

### https://en.wikipedia.org/wiki/Givaudan

This Wikipedia entry explains that Givaudan is one of the largest companies in the flavor and fragrance space. Like many of its competitors, the company traditional used synthetic chemistry but currently is increasingly using microbial biotechnology to make important molecules.

## Flavor and fragrance molecules

### https://www.sigmaaldrich.com/US/en/products/chemistry‐and‐biochemicals/flavors‐and‐fragrances

This commercial site of Sigma‐Aldrich is useful in explaining the different categories of flavor and fragrance molecules. It also has lists of chemicals in each category so one can learn the compounds that are important in the industry.

